# When Activism Becomes Survival: The Mental Health Costs of Constant Resistance in the Digital Era in the Balkans—A Health Policy Perspective

**DOI:** 10.3390/jmahp14020019

**Published:** 2026-04-02

**Authors:** Aleksandar Sič, Svetozar Mijuskovic, Nebojsa Brezic

**Affiliations:** 1 Faculty of Medicine, University of Belgrade, 11000 Belgrade, Serbia; svetozarmijuskovic3@gmail.com; 2 Department of Medicine, NYCHH/Lincoln, Bronx, NY 10451, USA; nebojsabrezic@gmail.com

**Keywords:** activism, mental health, health policy, access to care, health system governance, burnout, Balkans, health equity

## Abstract

Activism exposes individuals to sustained harassment, threat and psychological strain in contexts marked by discrimination and weak institutional protection. For LGBTQ communities, public engagement frequently increases vulnerability to both offline and digital harm, with cumulative consequences for mental health. Using the Balkans as a case example, this perspective sees activist mental health through a public health and health policy lens, framing distress not as an individual coping failure but as an outcome of structural barriers and minority stress processes, including inadequate legal protection, limited access to culturally competent mental health care and insufficient accountability for platform-mediated harm. This article highlights the population-level implications of unaddressed structural stressors, like burnout, disengagement and reduced sustainability of civil society participation, by situating activist mental health within broader questions of health system performance, access to care and governance. Upstream policy responses that strengthen institutional protection, ensure equitable access to mental health services and promote safer digital environments would address these challenges, positioning activist mental health as a critical public health policy issue.

## 1. Introduction

“Courage doesn’t always roar. Sometimes courage is the quiet voice at the end of the day saying, ‘I will try again tomorrow’.”

—Mary Anne Radmacher

The quiet persistence captured in this quote reflects the everyday reality of many activists operating in hostile sociopolitical environments, where sustained engagement often unfolds under conditions of chronic pressure and risk.

Activism in marginalized communities is rarely a free choice. In discriminatory and restrictive environments, it often becomes a matter of survival rather than political engagement [1]. For many LGBTQ individuals, especially transgender people, living openly itself is a form of resistance. Visibility carries risks and advocacy often reflects the need to defend basic dignity and safety rather than ideology [2].

While activism can foster solidarity and meaning, sustained engagement in hostile environments is linked to poorer mental health. High levels of anxiety, depressive symptoms and emotional exhaustion have been reported among activists exposed to sustained political pressure and threat [3,4]. Human rights activists commonly report chronic stress, emotional exhaustion and trauma-related symptoms due to ongoing exposure to threat and responsibility. This burden does not reflect individual vulnerability or lack of resilience, but arises from structural conditions that normalize discrimination, exclusion and harm [2,3], consistent with established conceptual frameworks on minority and structural stress in marginalized populations [5,6].

Today, much of this exposure is mediated through digital environments. Online platforms are now the main spaces for visibility, mobilization and community formation, especially where offline organizing is constrained. At the same time, digital spaces extend harassment, intimidation and surveillance into private life, blurring boundaries between activism and personal safety. For activists operating in hostile sociopolitical climates, this continuity of exposure limits opportunities for recovery and contributes to sustained psychological strain [7].

In this perspective, we want to argue that the mental health burden associated with human rights activism, particularly in the context of LGBTQ advocacy where personal identity and public engagement often overlap, should be understood as a public health issue shaped by structural violence, social exclusion and chronic exposure to threat. Shifting the focus from individual coping to the broader social, legal and digital environments that produce harm allows more effective and ethically grounded responses. Using the Balkans as a case example, we show how activism can become inseparable from survival and why protecting activist mental health is essential for both individual well-being and the long-term sustainability of civic engagement. This perspective draws on a critical synthesis of interdisciplinary literature to examine activist mental health from a health policy perspective. In this framework, activist distress is conceptualized as a downstream outcome of structural exposure to discrimination, digital hostility and limited institutional protection.

## 2. Digital Activism, Online Harm and Minority Stress

Digital platforms have become central infrastructures for activism where offline organizing is difficult or unsafe. For LGBTQ communities, they offer visibility and connection but also expose activists to constant harm and stress [8,9,10].

LGBTQ individuals are disproportionately targeted by online harassment, hate speech and intimidation, and repeated exposure to hostility is associated with poorer mental health outcomes (elevated anxiety levels, depressive symptoms and suicidal ideation). Estimates of cyberbullying victimization among LGBTQ youth vary widely across studies, ranging from approximately 10% to over 70%, with such exposure consistently linked to poorer mental health outcomes, including depressive symptoms and suicidal ideation [11]. For activists, this burden is often intensified. Public visibility also increases vulnerability to coordinated harassment campaigns, doxxing, defamatory narratives and threats. When institutional protection is limited, concerns about surveillance, retaliation or offline consequences of online activity are real rather than hypothetical [12,13,14].

Digital activism does not necessarily replace episodic offline engagement, nor is it inherently continuous; however, digital environments tend to extend exposure to hostility in more persistent and boundary-blurring ways, as harassment and hostile discourse can occur asynchronously and intrude into private spaces beyond specific moments of public advocacy. Consequently, online hostility is not limited to specific moments or spaces; it can follow activists into their private time and personal environments [15]. The persistent flow of negative messages, threats and distressing content creates a heightened state of alertness and reduces opportunities for psychological recovery. Over time, this continuous exposure contributes to emotional exhaustion, attentional fatigue and disengagement [16].

Many activists adopt protective strategies, some of which are: pseudonymous participation, self-censorship or the management of multiple online identities [17,18,19]. While these practices may reduce immediate harm, they require constant monitoring and emotional regulation. The cognitive and psychological effort involved in maintaining safety under conditions of visibility represents an additional layer of stress rather than a sustainable solution [17,18,19].

From a public health perspective, online harassment, surveillance and intimidation should be understood as structurally produced stressors rather than individual challenges. Digital hostility acts as an extension of minority stress, adding to the psychological burden created by offline discrimination. Its mental health impact cannot be addressed through personal resilience alone. Platform accountability, digital privacy protection, action against online hate and access to LGBTQ-affirmative mental health care are important components of prevention. In challenging sociopolitical environments such as the Balkans, ignoring digital harm negatively affects both activist well-being and the sustainability of civic engagement [20,21].

Health systems, regulators and digital platforms must coordinate responses to limit exposure to online abuse and safeguard activist well-being.

## 3. Activism as Survival in the Balkans

In the Balkans, efforts to claim basic rights, dignity or social recognition often take place in sociopolitical settings where such demands are seen as challenges to dominant cultural, religious or national norms. As a result, activities that would be considered routine civic participation elsewhere often carry greater personal risk [22]. The Balkan region is not politically or institutionally uniform, however, encompassing EU member states, candidate countries and complex post-conflict political systems; this discussion therefore refers to shared structural patterns, rather than identical national contexts.

Although several Balkan states formally recognize equality, implementation is often inconsistent and institutional protection is overall weak. Mechanisms for preventing or prosecuting hate-motivated incidents are limited or non-existent, and accountability is uneven. Public LGBTQ events regularly require extensive security measures or face restrictions justified on grounds of public order, showing the gap between rights on paper and lived safety [23].

Negativity towards LGBTQ communities in the region is often sustained by the circulation of nationalist, religious and anti-gender discourses that portray sexual and gender diversity as foreign, morally threatening or incompatible with national identity, reflecting locally specific expressions of broader risks faced by visible activists in restrictive sociopolitical environments. When these narratives appear across politics, media and public institutions, discrimination becomes normalized and sometimes implicitly legitimized. For LGBTQ individuals, this environment reinforces ongoing social exclusion and the sense that acceptance is fragile and can easily be withdrawn.

Within these conditions, activism often becomes inseparable from everyday self-protection. Activists must continuously assess risk, adapt behavior and anticipate potential threats in both offline and digital spaces. Limiting public visibility, modifying daily routines or maintaining partial anonymity online reflect not only caution, but the reality of navigating environments where personal safety is uncertain.

## 4. The Psychological Toll of Constant Resistance

### 4.1. Cumulative Trauma, Anxiety and Depressive Distress

As mentioned, activism under conditions of persistent pressure exposes individuals to repeated experiences of threat, intimidation and uncertainty. These exposures may be direct (harassment, violence or explicit threats) or indirect (continuous engagement with testimonies of abuse, hostile media narratives or distressing online content). Rather than occurring as isolated events, these stressors accumulate over time and produce the activation of psychological stress responses [24].

Trauma-related symptoms, anxiety and depressive distress frequently coexist. Activists report heightened vigilance, intrusive thoughts, sleep disturbances and persistent feelings of insecurity that extend beyond specific incidents. Chronic anticipation of harm and repeated public targeting can entrench these symptoms beyond specific incidents [25]. These outcomes are best understood not as just psychiatric disorders, but as predictable responses to prolonged exposure to negative surroundings. Thus, cumulative stress appears to be the primary driver of psychological burden. This comparison does not pathologize activism but shows that prolonged engagement under threat has mental health effects beyond the individual’s coping capacity.

### 4.2. Burnout, Hypervigilance and Emotional Depletion

Beyond trauma-related distress, sustained role demands in activism are associated with burnout and characterized by emotional exhaustion, reduced efficacy and detachment from previously meaningful work. When advocacy requires continuous availability, rapid responses to crises, and sustained emotional labor, opportunities for recovery become limited. Over time, this imbalance increases vulnerability to disengagement and withdrawal [26].

Hypervigilance often accompanies this process. The persistent monitoring of one’s surroundings, online presence and emotional expression may initially serve a protective function but becomes psychologically costly when maintained indefinitely. Activists frequently engage in ongoing emotional regulation to navigate scrutiny, hostility and expectations of moral consistency. This work demands sustained cognitive and affective effort, contributing to fatigue and diminished resilience [16,26].

In combination, burnout and hypervigilance reflect not individual failure, but structural conditions in which excessive demands coexist with insufficient protection and support.

### 4.3. Population-Level Mental Health Effects of Sustained Protest and Mobilization

Beyond individual symptom profiles, population-level mental health patterns observed during periods of sustained protest and social unrest indicate a broader epidemiological burden associated with collective political mobilization. Communities exposed to large-scale protests and riots exhibit elevated rates of trauma-related symptoms, with estimates of post-traumatic stress disorder ranging from approximately 4% to over 40%, alongside measurable increases in depressive symptomatology following major protest events. These effects extend beyond direct participants, suggesting spillover impacts on community mental health in environments characterized by prolonged instability and heightened social tension [27].

At the same time, longitudinal patterns indicate that the mental health consequences of civic engagement are not uniform. In electoral contexts, higher levels of pre-election activism have been associated with subsequent reductions in depressive symptoms and improvements in sleep quality, but only among individuals with strong identification with the political cause they supported. This heterogeneity suggests that activist engagement operates as a context-dependent exposure shaped by perceived legitimacy and alignment with collective goals [28].

## 5. When Activist Burnout Becomes a Population-Health Problem

A public health framing shifts the focus from individual coping to structural exposure to violence, discrimination and chronic social threat. The situation in Serbia reflects this dynamic, as the weak integration of human rights principles within institutional and healthcare systems contributes to predictable health disadvantages among vulnerable groups [29]. From this point of view, harmful environments are direct drivers of psychological distress rather than neutral background conditions. This perspective is particularly relevant in the Balkan context, where stigma is not episodic but persistent and visibility often increases rather than reduces risk. In Serbia, lesbian, gay and bisexual individuals report significantly higher levels of depressive symptoms and higher rates of suicide attempts compared to heterosexual participants [30].

Stigma related to both LGBTQ identities and mental health problems can discourage or delay seeking professional help. In many European health systems, access to mental health services is further constrained by underfunding, workforce shortages and uneven geographic distribution, so formal coverage does not always translate into real, timely care [31]. Beyond these structural capacity constraints, the stigma surrounding mental health disorders creates an additional barrier to care. Many people experiencing psychological distress delay or avoid seeking professional help because they fear labeling, discrimination or negative social consequences [32]. Stigma also shapes interactions within general medical settings, where sexual and gender minorities frequently report negative experiences with healthcare providers and may avoid both mental and somatic health services due to anticipated discrimination or breaches of confidentiality [33,34]. Across Europe, about one in five LGBTQ individuals experience discrimination in healthcare settings, reflecting ongoing barriers to equitable care [35]. As a result, many individuals postpone care or avoid it altogether, allowing mental health problems to worsen and leaving substantial needs unmet [32]. For activists, fears of discrimination or confidentiality breaches within healthcare settings may further reduce service use and weaken trust in health institutions. Unmet mental health needs among activists should therefore be understood not only as a psychosocial burden, but also as a matter of healthcare market access, reimbursement policies and equitable service availability within broader health system governance frameworks [33,36]. From a health systems perspective, these barriers correspond to well-established dimensions of healthcare access, including the availability, affordability, acceptability and appropriateness of services, as conceptualized in the access-to-care framework proposed by Levesque et al. [36].

Activists in these communities face an additional layer of exposure. Public-facing advocacy amplifies exposure to hostility, harassment and surveillance, turning activism into a sustained occupational risk. The psychological consequences observed among activists are comparable to those documented in other high-risk professions characterized by chronic exposure to threat and moral responsibility [37].

The effects of activist distress extend beyond individual well-being. Burnout and psychological withdrawal weaken organizational capacity, disrupt continuity of advocacy and erode informal support networks within marginalized communities. As experienced activists disengage, responsibilities concentrate among fewer individuals, intensifying exposure and accelerating cycles of exhaustion [26,38]. In this way, unaddressed activist distress becomes a systems-level failure with consequences for civil society resilience, democratic participation and public trust.

Personal coping and peer support remain valuable but cannot compensate for environments that continuously reproduce harm. Effective prevention requires structural interventions, including the reliable prosecution of hate-motivated violence, institutional protections for freedom of expression and assembly, safer digital environments and access to culturally competent, LGBTQ-affirmative mental health care. Without these measures, activist burnout should be understood not as an unfortunate side effect, but as an expected outcome of sustained engagement in harmful environments [26,37,39,40]. The importance of institutional context is also visible across Europe: differences in country-level structural stigma account for around 60% of the variation in life satisfaction among sexual minorities across 28 European countries, highlighting the health impact of legal and social environments [41].

Recognizing activist mental health as a population health issue shifts responsibility from individuals to the social, legal and political systems that shape exposure to risk. In the Balkans, where digital and offline pressure often overlap, ignoring these upstream factors affects both activist well-being and the sustainability of human rights advocacy.

## 6. Resilience, Community and Structural Preconditions for Sustainable Activism

Despite ongoing exposure to harm, many activists do show resilience. Their resilience is not a personal trait, but a product of community support, shared purpose and collective action. In this context, resilience should be understood as a protective capacity that helps individuals endure harmful environments, rather than as a substitute for structural protections. Solidarity and peer support can reduce psychological strain and help individuals endure hostile conditions. For some, activism itself restores a sense of control in environments designed to produce fear and marginalization [42,43].

However, resilience should not be idealized or treated as a solution to structural sources of harm. When harmful conditions persist, relying on individual endurance risks normalizing harm and shifting responsibility away from institutions. Activist mental health cannot be protected through self-care alone. Without legal protection, safer digital spaces and accessible, culturally competent mental health services, resilience becomes limited and uneven.

Sustainable activism requires environments that do not demand constant psychological sacrifice. Activist well-being should be treated as a core component of advocacy infrastructure, alongside legal protection and digital safety. When these conditions are absent, burnout increases and civic engagement weakens. At the population level, these implications point to coordinated responsibilities across health systems, regulatory bodies, digital platforms and educational institutions:

(1) legal and institutional protection, through the consistent prosecution of hate-motivated harassment and violence and safeguards for freedom of expression and assembly, given evidence linking weak enforcement to sustained minority stress and mental health disparities [5,6,23,30].
(2) safer digital environments, including platform accountability for harassment, doxxing and coordinated abuse, as well as protections for privacy and anonymity, as it has been shown that digitally mediated hostility directly contributes to psychological strain among LGBTQ activists [7,12,13,21].
(3) accessible, culturally competent and trauma-informed mental health services, delivered by professionals trained and sensitized to the specific stressors faced by activists and marginalized populations [37,38].
(4) preventive education and capacity-building, including the integration of human rights, digital safety and mental health literacy into formal education systems, alongside targeted, workshop-based training for activists, educators, journalists and public-sector professionals, given evidence that structural stigma and limited institutional awareness contribute to minority stress and health disparities [5,6,29].

**Figure 1:**
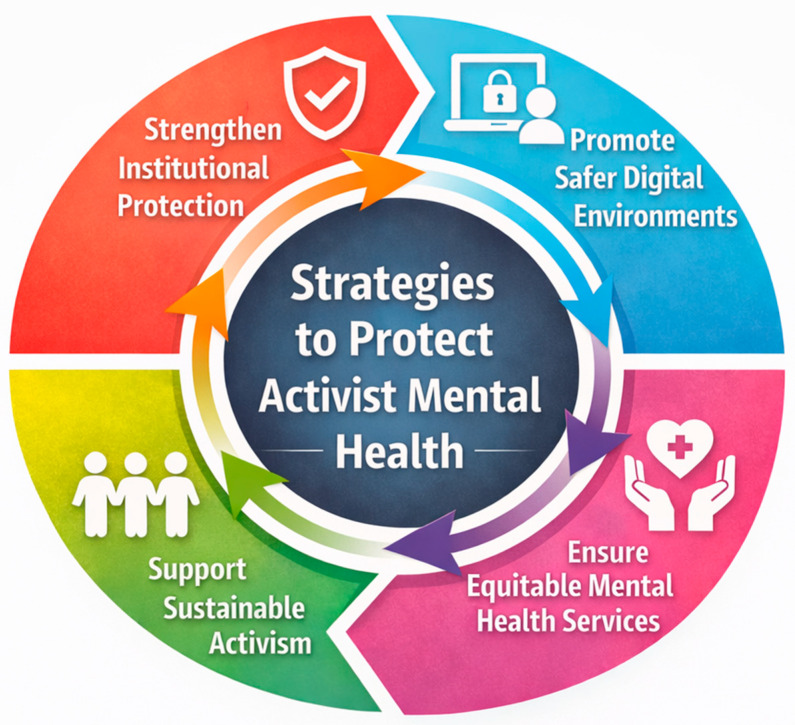
Conceptual framework of mentioned structural preconditions for sustainable activism and activist mental health protection. Created by the authors using Inkscape (version 1.0.1), an open-source vector graphics editor. Available at https://inkscape.org (accessed on 17 February 2026).

The key structural preconditions required for sustainable and mentally safe activism are summarized below in Figure 1.

This perspective relies on the existing literature and focuses on structural and digital factors without presenting new empirical data. Activists experience these challenges differently across the diverse Balkan region, but studies often use small, context-specific samples. Research also leaves significant gaps in understanding individual coping, and published work may emphasize extreme or highly visible cases. The patterns discussed here should be interpreted with attention to context and variation.

Failing to address activist mental health should be understood as a governance failure, not an individual outcome. As such, activist mental health should be recognized as a health policy and access issue, shaped by health system governance, service availability and regulatory responses to structural and digital forms of harm. Ensuring that courage does not depend solely on quiet endurance but is supported by protective systems is a measure of institutional and democratic maturity.

## Data Availability

No new data were created or analyzed in this study.
